# Prevalence of Unwanted Loneliness and Associated Factors in People over 65 Years of Age in a Health Area of the Region of Murcia, Spain: HELPeN Project

**DOI:** 10.3390/jcm13185604

**Published:** 2024-09-21

**Authors:** María Jesús Hernández-López, Solanger Hernández-Méndez, César Leal-Costa, Antonio Jesús Ramos-Morcillo, Isidora Díaz-García, María Verónica López-Pérez, Jessica García-González, María Ruzafa-Martínez

**Affiliations:** 1 Faculty of Social and Health Sciences, University of Murcia, Av. de las Fuerzas Armadas, 30800 Lorca, Spain; mariajesus.hernandez2@um.es (M.J.H.-L.); isidora.diaz@um.es (I.D.-G.); mariaveronica.lopez@um.es (M.V.L.-P.); 2 Faculty of Nursing, University of Murcia, Av. Buenavista 32, El Palmar, 30120 Murcia, Spain; ajramos@um.es (A.J.R.-M.); maruzafa@um.es (M.R.-M.); 3 Faculty of Health Sciences, University of Almeria, Carr. Sacramento, s/n, La Cañada, 04120 Almería, Spain; jgg145@ual.es

**Keywords:** loneliness, social isolation, aged, social support, depression, cognitive dysfunction

## Abstract

**Background/Objectives:** Population aging poses many challenges to public health, highlighting loneliness and social isolation as severe problems that affect the physical and mental health of older adults. During the COVID-19 pandemic, these became aggravated. The objective of the present study was to assess the prevalence of loneliness and its relationship with social isolation, depression, cognitive deterioration, sleep quality, and the level of physical mobility and functioning of older adults in Health Area 3 of the Region of Murcia. **Methods:** A descriptive, observational, and cross-sectional study was performed. The inclusion criteria were age ≥ 65, living in Health Area 3 of the Region of Murcia, and not being institutionalized. The following variables were evaluated: sociodemographic variables, loneliness (UCLA scale), social isolation (DUFSS), depression (GDS), cognitive deterioration (Pfeiffer), sleep quality (PSQI), and mobility (Barthel index). A univariate and multivariate regression model was created to examine how the dependent variable was related to the independent variables. **Results:** A total of 102 older adults participated in the study. Of these, 31.4% perceived unwanted loneliness and 14.7% low social support. The multivariate regression analysis showed that social isolation, geriatric depression, and cognitive deterioration were significant predictors of loneliness. **Conclusions:** The findings highlight the importance of developing multifaceted interventions that address not only social isolation but also other interrelated factors such as depression, cognitive deterioration, and sleep quality. The strategies should be centered on community programs and support networks. It is fundamental to perform longitudinal studies to better understand the causal relationships between these variables.

## 1. Introduction

In Spain, more than 20% of the population is over 65 years old. In Europe, this figure ranges from 23% in Italy; to 22% in Greece, Portugal, and Germany; to 14% in Ireland. It is estimated that in the next decade, those over 65 will exceed 30% of the population [1]. Population aging poses many challenges to public health [2]. Among them, loneliness and social isolation have emerged as severe problems that affect the physical and mental health and the quality of life and well-being of the elderly [3,4]. In addition, the effects of the COVID-19 pandemic must be considered, as the movement restrictions placed during that period of time increased the levels of loneliness and other negative emotional and cognitive symptoms [5,6].

In this sense, loneliness is defined as a subjective feeling of isolation or lack of company, despite the number of social contacts that a person may have; it is the perception of emotional and social disconnection. On the other hand, social isolation refers to the objective lack of social contacts and participation in social activities. Although related, these concepts are different: One is subjective and emotional, while the other is objective and structural. Current research shows that loneliness and social isolation are interrelated problems [7,8,9]. 

Approximately 43% of older adults in the United States experience loneliness [10], while 24% experience social isolation [11]. In European countries, the loneliness figures oscillate between 11.6% and 56%, with a higher prevalence in Mediterranean countries than in Northern European countries [12]. It is calculated that the percentage of adults with social isolation varies from 9% to 22% in Southern Europe, compared to 13% to 25% in Northern or Central Europe [13]. More specifically, in Spain, individuals 65 years old or older experience some type of loneliness, with a prevalence of between 41.3% and 44% in rural areas [14,15] and 35% in urban areas [16]; concerning social isolation, figures around 25.0% [17] and 25.98% [18] have been found.

Previous studies have shown that loneliness significantly contributes to depression and the cognitive deterioration of older adults [19,20]. Social isolation is linked to lower physical activity and worse sleep quality, which exacerbate feelings of loneliness [21]. Also, isolation is associated with severe health problems, including depression, a decrease in the quality of sleep, and a reduction in physical activity [10,11,12,13,14,15,16,17,18,19,20,21,22], and with a higher risk of suffering from cardiovascular diseases and premature death [10,11,12,13,14,15,16,17,18,19,20,21,22,23], as well as the risk of suicidal behavior and the likelihood of developing dementia syndrome [24]. Sociodemographic factors such as living alone or having low social support have also been associated with loneliness and social isolation in older adults [25]. Additionally, loneliness has been described as a mediating mechanism between elder abuse (both overall and in the forms of emotional or psychological abuse and neglect) and depressive symptoms, highlighting the need to address both loneliness and elder abuse in therapeutic and preventive interventions to alleviate depressive symptoms in older adults affected by these experiences [26]. Cultural and cross-cultural differences in loneliness and social isolation among older adults have also been observed [24]. Tomaka et al. compared Caucasian and Hispanic samples. According to their research, Hispanics are more likely to experience loneliness and develop health-related issues such as diabetes and hypertension. They also stated that having a sense of belonging to a place and having a source of support could be protective and eliminate the risk of developing these diseases [27].

Despite the growing evidence of these associations, there is still an urgent need for more detailed research in specific contexts [28,29] that explores how the interaction between loneliness, social isolation, and other health conditions affects older adults. A solid understanding of the epidemiology of loneliness is essential for supporting the design and development of interventions and the planning of services [30]. This analysis is even more necessary after the COVID-19 pandemic so that more up-to-date studies can provide information on the magnitude of the problem in specific contexts, identify factors that affect the well-being of older adults, and increase the knowledge that may facilitate the development of strategies and interventions for more effective care. The report from the Loneliness Barometer in Spain states that it is necessary to quantify the extent of unwanted loneliness in order to raise awareness and address the issue [31]. In this sense, the present study aimed to evaluate the prevalence of loneliness in the population of adults older than 65 years residing in Health Area 3 of Lorca, located in the Region of Murcia, Spain, and to explore the relationship between loneliness and key variables such as social isolation, depression, cognitive deterioration, sleep quality, and the level of physical mobility and functionality. 

## 2. Materials and Methods

### 2.1. Design

A descriptive, observational, and cross-sectional study was performed with individuals older than 65 years in Health Area 3 of the Region of Murcia, Spain.

### 2.2. Sample and Setting

In Spain, each region is divided into different health areas that serve specific geographical zones. For instance, the Region of Murcia comprises nine health areas. Health Area 3 in the Region of Murcia, Spain, where the study was carried out, is one of the highest in population. It encompasses a university hospital with 286 beds and 10 primary care health centers. In 2024, it provided services to a total population of 193,300 residents, including 29,897 individuals aged 65 years and older. 

A non-probabilistic sampling method was utilized, as subjects who were 65 years old or older were pre-selected by two means. Starting with patients in Health Area 3 in the Region of Murcia (Lorca) who were 65 years old or older, the nursing coordinators in the primary care health centers were contacted to solicit their collaboration in the pre-selection of eligible subjects. Through collaboration with the Poncemar Foundation and their volunteering program with older adults, “Banco Poncemar de Voluntariado” collaborates with the Department of Older Adults of the City Hall of Lorca. To ensure the sample’s representativeness, the sample size was calculated using Pass 11 (Power Analysis & Sample Size) software for multiple regression [32]. A sample size of 100 achieves 97% power to detect an R-squared of 0.20 attributed to 5 independent variable(s) using an F-test with a significance level (alpha) of 0.05.

The inclusion criteria were (1) being 65 years old or older, (2) living in Health Area 3 in the Region of Murcia, and (3) not being institutionalized. The following individuals were excluded: (1) those with severe cognitive deterioration (8–10 mistakes in the Pfeiffer test—SPMSQ); (2) those who had difficulties in answering the measurement scale due to a language barrier; (3) those having any type of physical, mental, or legal disability, which, to the researcher’s discretion, may impede completing the measurement instruments or participating in the study; and (4) those who did not grant consent to participate in the study.

This volunteer program serves individuals aged 65 and over residing in Lorca and recruits participants through social services staff. The social services technicians from the Lorca City Council conducted a social assessment (evaluating the available human and material resources in the home, the degree of autonomy of the older adult, etc.) of the participants prior to their selection and inclusion in the “Poncemar Volunteer Bank”.

The study population consisted of 102 individuals over the age of 65 with no prior diagnosis of loneliness or social isolation and not residing in institutions.

### 2.3. Variables and Measurement Instruments

The study took into account the following variables:

(1) Sociodemographic characteristics of the participants: age, sex, marital status, level of education, number of children, last employment, amount and type of income, rural or urban setting, living alone or with someone and, in this case, the number of people.

(2) Loneliness was evaluated with the revised loneliness scale from the University of California, Los Angeles (UCLA) [33]. This questionnaire comprises ten items and is evaluated through a Likert scale with four response options. It is scored from 1 to 4, and scoring oscillates between 10 and 40 points. A score < 20 indicates a severe degree of loneliness, and a score between 20 and 30 indicates moderate loneliness. The UCLA scale has a construct validity with high correlations between the items, adequate discriminant validity, and high reliability according to a Cronbach’s alpha of 0.95.

(3) Social isolation was measured with the social support questionnaire Duke-UNC (DUFSS) [34]. This questionnaire is composed of 11 items and is evaluated with a Likert scale of 5 response options ranging from 1 to 5, with a final score that oscillates between 11 and 55 points. The score obtained indicates the social support perceived, with a lower score indicating less support (a score ≥ 32 indicates normal support; a score < 32 indicates low perceived support). The Cronbach’s α score was 0.94, and the factorial analysis showed two factors on both scales with an explained variance of 73.8%.

(4) Depression was evaluated with the geriatric depression scale (GDS) [35]. This questionnaire is composed of 15 items and is utilized for the screening of depression in individuals older than 65. The subjects must answer with “Yes” or “No.” Ten items indicate the presence of depression symptoms if they are answered affirmatively, while the remaining 5 indicate symptoms of depression if answered negatively. The score varies from 0 to 15 points, and 1 point is given to each response that indicates the presence of symptoms of depression. The categorization based on the scale’s score is 0–4 points: normal (no symptoms of depression), 5–8 points: light symptoms of depression, 9–10 points: moderate symptoms of depression, and 12–15 points: severe symptoms of depression. The GDS obtained a sensitivity of 0.82, a specificity of 0.8, a positive predictive value of 0.86, a negative predictive value of 0.97, and a positive likelihood ratio of 35.03.

(5) Cognitive deterioration was evaluated with the Pfeiffer Short Portable Mental Status Questionnaire (SPMSQ) [36]. This questionnaire comprises ten questions that detect the existence and degree of cognitive deterioration, evaluating short-term memory and orientation. The questions the participant answers incorrectly are marked, with the final score added at the end (each mistake equals one point) so that the score varies between 0 and 10 points. An extra error is allowed if the participant did not receive primary school education, and one less error if the participant had received higher education. The categorization based on the score obtained on the scale is 0–2 mistakes: normal (no cognitive deterioration), 3–4 mistakes: light cognitive deterioration, 5–7 mistakes: moderate cognitive deterioration, and 8–10 mistakes: severe cognitive deterioration. The inter- and intra-observer reliabilities were 0.738 and 0.925, respectively, with the internal consistency reaching a value of 0.82. The convergent validity was 0.74, and the discrimination was 0.23. The area under the ROC curve was 0.89, with the sensitivity and specificity being 85.7 and 79.3, respectively, for a cut-off point of 3 or more mistakes, with variations observed according to the level of education and age.

(6) Quality of sleep was measured with the Spanish version of the Pittsburgh Sleep Quality Index (PSQI) [37]. The Pittsburg Sleep Quality Index contains 19 items grouped into 10 questions that assess sleep habits in the previous month. The 19 questions are combined to obtain seven areas with corresponding scores, with each ranging between 0 and 3 points (subjective quality, duration, latency, efficiency, disorders, use of medication for sleeping, and daytime dysfunction), which are totaled at the end to obtain an overall score, which oscillates between 0 and 21 points (0 indicates easiness falling asleep, and 21 indicates a severe difficulty in all areas). The instrument had an internal consistency (measured with Cronbach’s alpha) of 0.81, a positive Kappa coefficient of 0.61, a sensitivity of 88.63%, a specificity of 74.99%, and a positive predictive value of 80.66.

(7) Level of mobility, functionality, and physical state assessed with the Barthel index [38]. This instrument allows us to measure the ability of a person to perform ten activities of daily life considered to be basic to obtain a quantitative estimation of their degree of dependence. The participant is interrogated about each of the activities, and according to their ability to perform them, they are given a score of 0, 5, or 10 (15 in specific activities), with a maximum score of 100 points. The categorization, based on the score from the scale, is 0–20: total dependence, 25–60: severe dependence, 65–90: moderate dependence, 95: slight dependence, and 100: independence. The Cronbach’s alpha coefficients were higher than 0.70. The confirmatory factor analysis showed satisfactory fit indices and factorial loads. The validity of known groups showed significant differences in the Barthel index according to age, the number of comorbidities, and sex.

All questionnaires were chosen because they are standardized, internationally validated, and widely used tools that have been satisfactorily adapted and validated in Spanish.

### 2.4. Data Collection

The data were collected during January and May 2023 through telephone interviews and was recorded in the ICEBE Digital Electronic History Registry platform created for the management of the HELPeN program. The data collected were sociodemographic and health variables. The clinical data remained separated from the identification data, and the databases were encrypted and kept safe in protected servers through access via a password. Only the main project researchers had access to the data through a password to maintain the confidentiality of the data. The remaining members of the research team had access to limited data, according to the activity that each performed.

This evaluation was part of the telephone-based telenursing HELPeN Program (Help e Nursing), which was directed toward managing social isolation in older adults [39].

### 2.5. Ethical Considerations

The study was conducted following the ethical principles of the Declaration of Helsinki and was authorized by the Research Ethics Committee from the University of Murcia (n. 3267/2021). It was also authorized by the Research Committee of the Health Area 3 Management of the Region of Murcia to have access to the data necessary for performing the study. All the participants were informed of the general aspects of the study and the right to withdraw their consent to participate at any time. Also, the participants signed an informed consent form before participating in the study. The participants were identified using a code number maintained throughout the study to avoid their identification and to guarantee confidentiality. The study met the requirements established by EU Regulation 2016/679 of the European Parliament and the Council from 27 April 2016 on the General Data Protection Regulation (GDPR) [40].

### 2.6. Data Analysis

The descriptive statistics summarized the participants’ characteristics and the scores of the measurement instruments. The categorical variables were expressed as frequencies and percentages. The mean and standard deviation (SD) were calculated for the quantitative ones.

Pearson’s r correlation coefficient was used to evaluate the relationship between the variables of interest. Lastly, a univariate and multivariate forward stepwise linear regression model was created to examine how the dependent variable (loneliness) was correlated with the independent variable (social isolation, level of mobility, functionality, physical state, cognitive deterioration, geriatric depression, sleep quality). To ensure the validity of the multiple linear regression analysis, we checked the normality assumption for the residuals using Q-Q plots.

The statistical analysis used a significance level of 5% (*p* ≤ 0.05). The analysis used SPSS (Statistical Package for the Social Sciences) v. 24.0.

## 3. Results

### 3.1. Description of the Participants

The final sample was composed of 102 participants (Table 1). The mean age was 75.76 (SD = 6.36) years, and 52.9% (n = 540) were women. Regarding the level of education, 69.6% did not have an education, 44.1% were married, 53.9% lived in a semi-urban setting (10,000 to <50,000 inhabitants), and 49% lived with one housemate/partner. Table 1 shows the main sociodemographic characteristics of the participants.

### 3.2. Results from the Measurement Tools

Table 1 shows the mean scores of the measurement instruments utilized. The participants obtained a low perception of loneliness and social isolation, as shown by the high scores on the scales, which indicate less loneliness and social isolation. On the other hand, when the variables were categorized, it was observed that 32 (31.4%) participants had a moderate–severe degree of loneliness. Concerning the social isolation variable, 14.7% (n = 15) had low perceived social support. For the rest of the variables, low scores were obtained in the variables of geriatric depression, cognitive deterioration, and sleep quality. In contrast, high scores were found for the participants’ mobility, functionality, and physical state.

### 3.3. Correlation and Regression Models of the Study Variables

The bivariate analysis showed a positive correlation with moderate–high and statistically significant coefficients between the loneliness and social isolation variables. This indicates that the greater the perceived social isolation, the higher the degree of loneliness perceived by the participants (Table 2).

On the other hand, a negative correlation was obtained with moderate–high and statistically significant coefficients between the variables of loneliness, social isolation, geriatric depression, cognitive deterioration, and sleep quality. This indicates that the lower the score in the variables of loneliness and social isolation (which indicate a greater perception of loneliness and social isolation), the higher the score in the variables of geriatric depression, cognitive deterioration, and sleep quality (Table 2).

Table 3 shows the univariate and multivariate linear regression model, with loneliness as the dependent variable and social isolation, level of mobility, functionality, physical state, cognitive deterioration, geriatric depression, and sleep quality as the independent variables.

In the univariate linear regression analysis, all the independent variables, except for mobility, functionality, physical state, and age, showed associations with a probability of error of less than 0.05 with respect to the dependent variable. The variance explained by the significant variables oscillated between 6.4% and 47.3%.

The multivariate linear regression model explained 51.3% of the variance (adjusted R^2^ = 0.513). The t-test revealed an association, with a probability of error of less than 0.05, for the variables included in the model: social isolation, geriatric depression, and cognitive deterioration. Also, the Durbin–Watson statistic pointed to the absence of self-correlation in the residues of the multiple linear regression model (D = 2.04). The tolerance and VIF values indicated the absence of multi-collinearity between the variables in this model.

**Figure 1:**
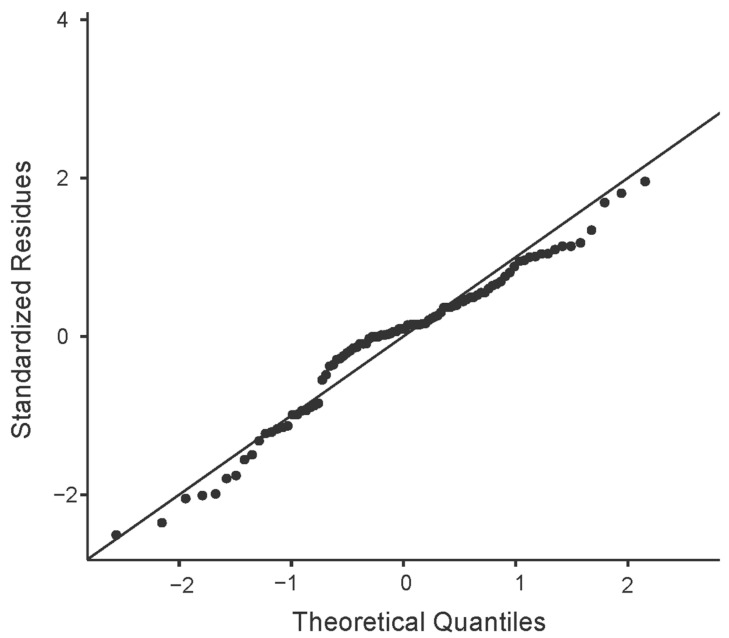
Q-Q plots of multiple linear regression analyses.

To ensure that the multiple linear regression analysis’s assumptions of normality were met, we visually inspected the residuals’ Q-Q plots. The plots demonstrated that the data points closely followed the reference line, indicating that the residuals were approximately normally distributed (Figure 1).

## 4. Discussion

The results from the present study highlight the prevalence of loneliness in a sample of adults older than 65 residing in Health Area 3 in the Region of Murcia, as well as the relationship between loneliness and diverse key variables such as social isolation, depression, cognitive deterioration, sleep quality, and the level of mobility and functionality. The typical study participant was a Spanish woman with an average age of 65, without formal education, and married or widowed. She primarily lived in semi-urban areas and tended to live with one or two others.

This profile underlines the need to consider sociodemographic factors in designing interventions to address loneliness and social isolation in the older adult population. In line with previous studies, gender differences have been identified, showing a higher prevalence of social isolation and loneliness among the female population [24]. In addition, the level of education indicates a possible barrier to access to information and resources. This link between the level of education and access to resources has been previously explored, indicating that higher levels of education facilitate greater access to information and social support networks, which reduces loneliness and improves quality of life [25]. On the other hand, although there was a high percentage of older adults who were married (44.1%) and who lived with someone (92%) in our study, it is known that the factors of loss of a partner and living alone are associated with higher levels of loneliness in previous studies [13,23,25]. Also, 14.7% lived in urban areas, 53.9% in semi-urban environments, and 31% in rural areas, so it can be considered that, on the one hand, the area of residence could influence the opportunities for social interaction and access to support services that could mitigate the impact of isolation [21,41,42]. In addition, living with others could provide social support and reduce loneliness, although it can also result in challenges if the relationships are conflictive or non-satisfactory [19,20,43]. Lastly, studies on family and co-living dynamics indicate that the quality of interpersonal relations plays a crucial role in how individuals experience loneliness [16].

In our study, the prevalence of loneliness with moderate to severe levels was 31.4%, a result that was found to be consistent with previous studies that reported similar rates in the population of elders [16,25,44]. Nevertheless, this figure is slightly lower than that reported by Gene et al., who indicated a prevalence of loneliness of 35.25% [16], and somewhat higher than the values reported by Su Y et al., who documented a prevalence of 28.6% [45]. In addition, the prevalence of social isolation in our study was 14.7%, lower than in other studies, which indicated a prevalence of 19% [46]. The differences in the rates of loneliness and social isolation with respect to other studies could be explained by various factors, including cultural differences, the specific point in time of the study, the demographic characteristics, and other characteristics of the sample, such as the state of health, the socio-economic level, the existing social support network or sentimentalism and nostalgia. A transcultural study conducted in Europe examines how sentimentalism and nostalgia in older adults may be linked to an increase in depression, especially due to a lack of support. These feelings are found to be related to loneliness, depression, and sleep quality [47]. Exploring these aspects could provide a deeper understanding of the underlying dynamics influencing loneliness and social isolation in different populations.

Our findings show significant correlations between loneliness and social isolation, indicating that the greater the perception of social isolation, the greater the perception of loneliness. Also, social isolation emerged as a stronger predictor of perceived isolation, explaining 47.3% of the loneliness variance. This result coincides with previous studies that showed that unwanted loneliness was perceived as a negative experience, and its increase was related to social isolation [3,14]. 

Likewise, a significant correlation was observed between loneliness and geriatric depression, which is consistent with previous studies that have demonstrated that loneliness is a significant risk factor for depression in older adults [10,22]. A systematic review highlighted that older adults with high levels of loneliness and social isolation were more prone to experiencing symptoms of depression and cognitive deterioration, thus pointing out that loneliness not only forecasts depression but also exacerbates the existing symptoms of depression [48].

Significant negative correlations were also found between loneliness and cognitive deterioration, suggesting that loneliness can worsen it, as shown in other studies [10,23,49,50,51]. The scientific literature supports these findings, indicating that loneliness is related to higher psychological and physical morbidity, including depression, cognitive deterioration, dementia, and other chronic diseases [42,52,53,54,55,56], and the onset of psychotic symptoms without prior neurological conditions [26]. Lastly, sleep quality showed a significant correlation with loneliness, which was not maintained in the multivariate analysis, and it is therefore believed that this variable could be linked with others, such as depression or cognitive deterioration. This result differs from previous studies that showed that loneliness negatively affects sleep patterns [7]. Thus, new studies must be conducted that delve into this relationship.

### 4.1. Implications and Future Directions

The results suggest the need for interventions to reduce loneliness in older adults and increase social support. Given the significant impact of mental health and general well-being, the strategies should be centered on promoting social support and opportunities for social interaction. Community programs, support networks, and volunteer programs could play a crucial role in the remission of these problems. Also, specific programs should be developed to address depression and cognitive deterioration, as these factors are also closely related to loneliness.

Future research must conduct longitudinal studies to better understand the causal relationships between these variables and how they change over time. The addition of objective measures of social interaction and mental health could help validate the results obtained through the use of self-completed questionnaires and reduce response bias.

### 4.2. Study Limitations

The present study has various limitations to be considered when interpreting the results. In the first place, the study’s cross-sectional design impeded the establishment of causal relationships between the variables. Although significant associations were identified, the direction of these relationships could not be determined. In second place, the sample was selected through a non-probabilistic method, which could limit the generalization of these results to other older adult populations. Using self-reported questionnaires and telephone interviews could introduce response biases, as participants might underestimate or overestimate their loneliness and social isolation levels. Telephone interviews have some disadvantages. Auditory difficulties, lack of familiarity with telephone devices, and poor signal quality can hinder communication. Additionally, memory issues can complicate following instructions, and the lack of non-verbal cues in a telephone interview makes interpretation more challenging. To address these issues, trained interviewers with expertise in administering the self-reported questionnaires were employed. Lastly, although diverse variables were included, it is possible that other factors that were not considered in this study also influence the loneliness and social isolation of older adults, such as nostalgia and sentimentalism, which are related to loneliness and are not examined in this study. In addition, there could be an influence of contextual factors that were not measured, such as changes in living conditions or the state of health during the study period.

## 5. Conclusions

This study provides a comprehensive view of the prevalence and factors associated with loneliness in older adults in the Region of Murcia. The findings highlight the importance of developing multifaceted interventions that address not only social isolation but also other interrelated factors, such as depression, cognitive deterioration, and sleep quality. These interventions could significantly improve these older adults’ well-being and quality of life, thus creating more effective and directed health policies.

## Figures and Tables

**Table 1:** Descriptive statistics on the characteristics of the sample.

Categorical Variables	*n*	*%*
Gender		
Male	48	47.1
Female	54	52.9
Nationality		
Spain	99	97
Ecuador	2	2
Argentina	1	1
Level of education		
None	71	69.6
Primary (EGB, primary, ESO)	22	21.5
Secondary (vocational, baccalaureate)	2	2.0
University	7	6.9
Marital status		
Single	11	10.8
Married	45	44.1
Divorced	3	2.9
Separated	6	5.9
Widowed	37	36.3
Environment		
Urban (equal to or more than 50,000 inhab.)	15	14.7
Semi-urban (10,000 a <50,000 inhab.)	55	53.9
Rural (<10,000 habt)	32	31.4
Number of cohabitants		
0	8	7.8
1	50	49.0
2	36	35.3
3	6	5.9
4	2	2.0
Quantitative variables	M	SD
Age (years)	75.76	6.36
No. of children	2.12	1.53
Loneliness (UCLA) (range 10–40)	34.41	6.18
<20 Severe degree of loneliness (n %)	2	2
20–30 Moderate loneliness (n %)	30	29.4
Social isolation (DUFSS) (range 11–55)	41.70	8.55
<32 Low perceived social support (n %)	15	14.7
≥32 Low perceived social support (n %)	87	85.3
Depression (GDS) (range 0–15 points)	2.40	2.08
Cognitive deterioration (SPSMQ)	0.81	1.28
0–2 Normal (n %)	90	88.2
3–4 Slight cognitive deterioration (n %)	11	10.8
5–7 Moderate cognitive deterioration (n %)	1	1.0
8–10 Severe cognitive deterioration (n %)	0	0
Sleep quality (PSQI) (range 0–21)	5.17	3.13
Level of mobility, functionality, and physical state (Barthel) (range 0–100)	96.78	7.54

**Table 2:** Bivariate correlations of the study variables.

	Age	DUK	BTH	GDS	PFE	PIT	UCLA
Age	1						
DUK	0.051	1					
BTH	−0.513 **	−0.002	1				
GDS	−0.040	−0.495 **	−0.279 **	1			
PFE	0.474 **	−0.084	−0.403 **	0.224 *	1		
PIT	0.071	−0.317 **	−0.232 *	0.529 **	0.241 *	1	
UCLA	0.040	0.692 **	0.160	−0.534 **	−0.271 **	−0.395 **	1

** The correlation is significant at 0.01 (two-tailed). * The correlation is significant at 0.05 (two-tailed). UCLA: loneliness; DUK: social isolation; BTH: functionality; GDS: geriatric depression; PFE: cognitive deterioration; PIT: sleep quality.

**Table 3:** Univariate and multivariate linear models.

Dependent Variable	Loneliness						
	Univariate				Multivariate		
Independent Variables	*β*	*t*	*p*-Value	R^2^ Adjust	*β*	*t*	*p*-Value
Social isolation (DUFSS)	0.692	9.58	<0.001	0.473	0.567	6.78	<0.001
Depression (GDS)	−0.534	−6.12	<0.001	0.277	−0.221	−2.58	0.012
Cognitive deterioration (SPSMQ)	−0.271	−2.79	0.006	0.064	−0.148	−1.98	0.050
Sleep quality (PSQI)	−0.395	−4.17	<0.001	0.147			
Level of mobility, functionality, and physical state (Barthel)	0.160	1.61	0.11	0.016			
Age (years)	0.040	0.40	0.69	−0.008			
					R = 0.727; adjusted R^2^ = 0.513; F = 34.004; *p* < 0.001; Durbin–Watson’s D statistic = 2.04		

*β* = standardized coefficients.

## Data Availability

The data presented in this study are available on request from the corresponding author due to ethical reasons.
